# Combined Effects of Curcumin and Lycopene or Bixin in Yoghurt on Inhibition of LDL Oxidation and Increases in HDL and Paraoxonase Levels in Streptozotocin-Diabetic Rats

**DOI:** 10.3390/ijms18040332

**Published:** 2017-03-23

**Authors:** Renata Pires Assis, Carlos Alberto Arcaro, Vânia Ortega Gutierres, Juliana Oriel Oliveira, Paulo Inácio Costa, Amanda Martins Baviera, Iguatemy Lourenço Brunetti

**Affiliations:** Department of Clinical Analysis, School of Pharmaceutical Sciences, São Paulo State University-UNESP, Araraquara, São Paulo 14800-903, Brazil; renatapires_17@hotmail.com (R.P.A.); carlos_arcaro@hotmail.com (C.A.A.); vania_orteg@hotmail.com (V.O.G.); juoriel@hotmail.com (J.O.O.); costapi@fcfar.unesp.br (P.I.C.); baviera@fcfar.unesp.br (A.M.B.)

**Keywords:** diabetes mellitus, curcumin, bixin, lycopene, oxidative stress, cardiovascular risk

## Abstract

Combination therapy using natural antioxidants to manage diabetes mellitus and its complications is an emerging trend. The aim of this study was to investigate the changes promoted by treatment of streptozotocin (STZ)-diabetic rats with yoghurt enriched with the bioactives curcumin, lycopene, or bixin (the latter two being carotenoids). Antioxidants were administered individually, or as mixtures, and biomarkers of metabolic and oxidative disturbances, particularly those associated with cardiovascular risk, were assessed. Treatment of STZ-diabetic rats with natural products individually decreased glycemia, triacylglycerol, total-cholesterol, oxidative stress biomarkers, including oxidized low-density lipoprotein (ox-LDL), and increased the activities of antioxidant enzymes. Individual carotenoids increased both high-density lipoprotein (HDL) and paraoxonase levels, whereas curcumin increased only paraoxonase. Treatments with mixtures of curcumin and lycopene or bixin had combined effects, decreasing biomarkers of carbohydrate and lipid disturbances (curcumin effect), increasing the HDL levels (carotenoids effects) and mitigating oxidative stress (curcumin and carotenoids effects). The combined effects also led to prevention of the LDL oxidation, thereby mitigating the cardiovascular risk in diabetes. These findings provide evidence for the beneficial effect of curcumin and carotenoid mixtures as a supplementation having antioxidant and antiatherogenic potentials, thus appearing as an interesting strategy to be studied as a complementary therapy for diabetic complications.

## 1. Introduction

Diabetes mellitus (DM) is a chronic endocrine syndrome resulting from a deficiency in pancreatic insulin production and/or insulin resistance in target tissues, leading to various abnormalities in carbohydrate, lipid, and protein metabolism [1]. If uncontrolled, DM can lead to a variety of microvascular (diabetic nephropathy, retinopathy, and neuropathy) and macrovascular (atherosclerosis, coronary artery diseases, and stroke) complications; these are the major causes of morbidity and mortality in diabetes [2]. DM affects approximately 415 million people worldwide, and this is predicted to rise to 642 million by 2040 [3]. In many countries, changes in diet and lifestyle account for the epidemic in obesity and DM, especially type 2 DM [4]. The health costs associated with DM are very high; approximately 11% of the global health expenditure is directed to the treatment of DM and its related complications [5].

Individuals with DM have many of the risk factors associated with cardiovascular disease (CVD) [6]. CVD is the leading cause (approximately 70%) of death in people with DM [7]. A combination of various factors, including oxidative stress, endothelial dysfunction, and low-grade inflammation, accounts for the increased risk of CVD in people with diabetes [8]. Oxidative stress in DM originates from the increased production of reactive oxygen species (ROS) mostly arising from increased mitochondrial electron transport chain activity [9] caused by hyperglycemia. Oxidative stress in DM is widely accepted to be an important component in the production of oxidized LDL (ox-LDL) [10].

Ox-LDL, itself, is crucial for the development of atherosclerotic lesions [11,12]; the uptake of this modified lipoprotein occurs via scavenger receptors found on macrophages leading to the generation of foam cells, the hallmark of atherosclerotic lesions [13]. Ox-LDL also has various other pro-atherosclerotic effects, such as causing endothelial dysfunction via stimulation of superoxide anion radical (O_2_^•−^) production and smooth muscle vascular remodeling [14].

The search for effective strategies, using natural antioxidants, to prevent LDL oxidation and reduce CVD risk is an emerging trend [15]. A therapeutic intervention able to control hyperglycemia and which increases both HDL and the levels of paraoxonase 1 (PON1) could offer additional protection against long-term diabetic complications. An interesting approach that has been suggested is to create a combination therapy based on natural compounds derived from medicinal plants or functional foods to manage DM and its complications [16,17]. Underpinning this idea is the fact that, in fruits and vegetables, natural antioxidants exist in combination; they act synergistically as antioxidants and also provide other pharmacological properties, explaining the health benefits of these foods [18].

This study focused on three natural products, namely curcumin, lycopene, and bixin. Curcumin (from *Curcuma longa* L. rhizomes) is used in many food products and dishes, especially those spiced with turmeric, such as curry and yellow rice. Lycopene is found in tomatoes, watermelon, papaya, guava, and grapefruit. Bixin (from *Bixa orellana* L. seeds) is used as a colorant in a range of cosmetics and foods (butter, cheese, bakery products, oil, cereal, and sausage). A wide range of beneficial effects towards the metabolic disturbances associated with DM has been attributed to these natural antioxidants [19,20,21,22,23], motivating the study of combinations of these antioxidants as a complementary strategy for the prevention of long-term complications of diabetes. There is no data available, as far as we know, about the prospective in vivo benefit of these natural ingredients when used in combination.

Curcumin, lycopene, and bixin all have low solubility in water. To overcome this problem, yoghurt was chosen as the vehicle for the oral administration of these natural antioxidants in the in vivo study reported here. Previous in vivo studies have used vegetable oils when administering these compounds by oral gavage. However, considering that dyslipidemia is a feature of STZ-diabetic rats, and also that this study was performed to investigate the effects of these compounds on lipid metabolism, we elected to avoid using oil as the vehicle. Yoghurt was chosen as the vehicle for the oral administration of these natural antioxidants because of the current trend in the consumption of food matrices enriched with bioactives derived from functional foods and/or medicinal plants, so this appeared to be an interesting option to treat or prevent chronic diseases [24,25].

The aim of the present study was to investigate the changes promoted by the long-term treatment of STZ-diabetic rats with yoghurt enriched with curcumin, lycopene, or bixin, individually, or as mixtures, on various biomarkers related to the metabolic and oxidative disturbances observed in this experimental model of type 1 DM.

## 2. Results

### 2.1. Characterization of the Experimental Model of Type 1 Diabetes Mellitus (DM)

In this study, normal or diabetic rats treated with yoghurt (referred to as NYOG and DYOG, respectively) were considered the control groups related to the absence (NYOG) or the presence (DYOG) of metabolic disturbances arising due to insulin deficiency. The choice of yoghurt as a suitable vehicle for a study in DM was aided by a previous publication by Gutierres et al. [19]. They observed that there were no differences in physiological or biochemical parameters in normal rats treated with yoghurt compared with normal rats treated with water; the same was true for the comparison among diabetic rats. Thus, in the present study, yoghurt was selected as an inert vehicle for antioxidant administration.

**Figure 1:**
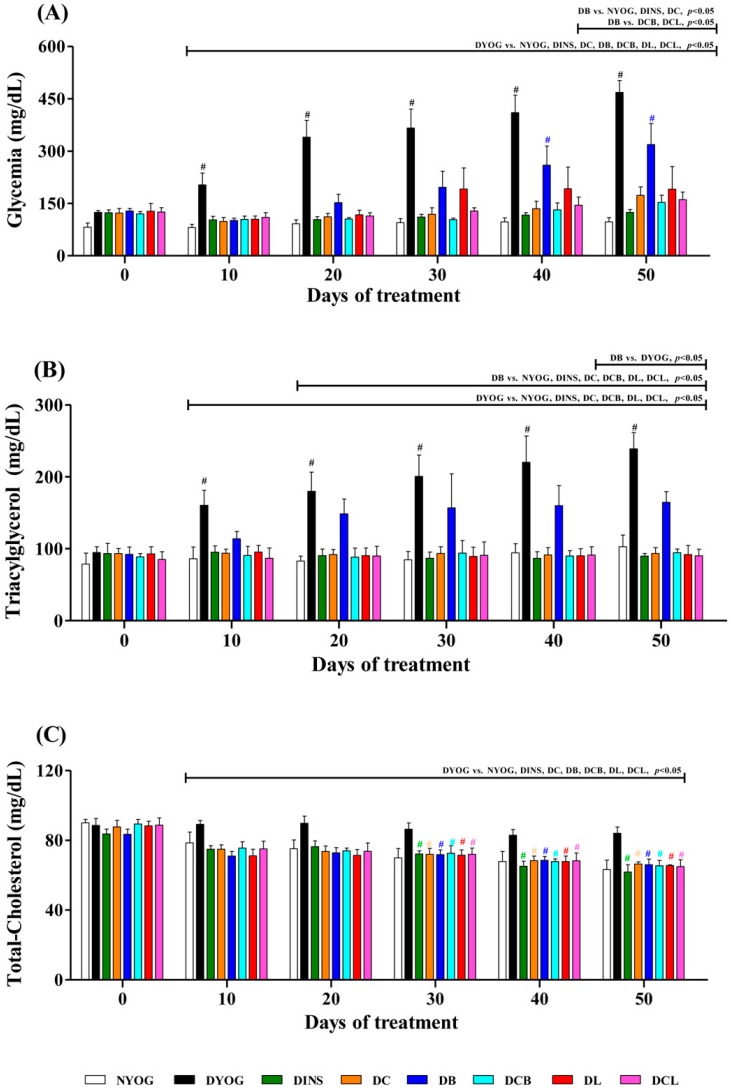
Temporal changes in glycemia (**A**); triacylglycerol (**B**); and total-cholesterol (**C**) plasma levels in STZ-diabetic rats treated for 50 days with yoghurt enriched with curcumin and carotenoids, individually or as mixtures. Values are expressed as means ± SEM, *n* = 10. Differences between groups were considered significant at *p* < 0.05 and were analyzed by one-way ANOVA followed by a Student-Newman-Keuls test. Differences in the same group relative to day 0 were analyzed using a paired Student’s t test. #, different compared to day 0 (*p* < 0.05).

The DYOG group showed the expected changes for a condition of insulin deficiency, typical of a type 1 DM experimental model. DYOG rats showed initial postprandial glycemia levels of approximately 450 mg/dL (3 days after STZ), similar to those in all the other diabetic rat groups prior to treatment, indicating that the induction of diabetes in rats was effective. Postprandial glycemia was used to confirm the establishment of DM and also to sort diabetic animals into equally matched groups prior to treatment. After the beginning of treatment, blood samples were collected, following a 12 h fast, every 10 days, for 50 days. At day 0, DYOG rats (5 days after STZ) had fasting glycemia levels of 128 mg/dL that, although greater than values of NYOG animals (82 mg/dL), were not statistically different (Figure 1A); however, fasting glycemia was progressively increased in DYOG rats (Figure 1A), evidencing the worsening of diabetes. In contrast, NYOG rats maintained glycemia within normal values over the course of the experiment (Figure 1A).

Reduced body weight gain, reduced terminal body weight (Table 1), as well as reduced adipose and skeletal muscle masses (Table 2), were observed in DYOG rats compared with NYOG, which is typical for the catabolic state caused by insulin deficiency. Other physiological and biochemical changes classically observed in this experimental model of type 1 DM were also observed in DYOG rats, such as polyphagia, polydipsia, and polyuria (Table 1). Dyslipidemia was also evident, with increased fasting plasma levels of triacylglycerol (Figure 1B) and total cholesterol (Figure 1C) being observed.

**Figure 2:**
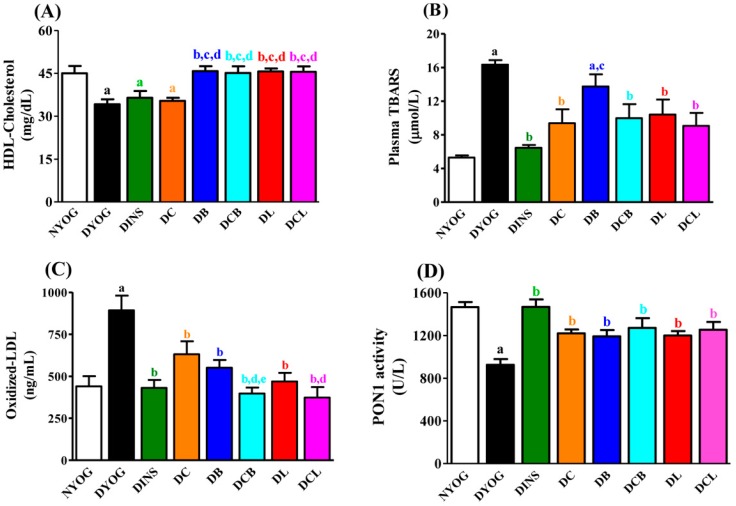
Plasma levels of high-density lipoprotein (HDL) cholesterol (**A**) thiobarbituric acid reactive substances (TBARS) (**B**); oxidized low-density lipoprotein (ox-LDL) (**C**); and paraoxonase 1 (PON1) activity (**D**) of STZ-diabetic rats after 50 days of treatment with yoghurt enriched with curcumin and carotenoids, individually or as mixtures. Values are expressed as means ± SEM, *n* = 10. Differences between groups were considered significant at *p* < 0.05 and were analyzed with one-way ANOVA followed by Student-Newman-Keuls test. a, different compared to NYOG; b, different compared to DYOG; c, different compared to DINS; d, different compared to DC; e, different compared to DB (*p* < 0.05).

**Figure 3:**
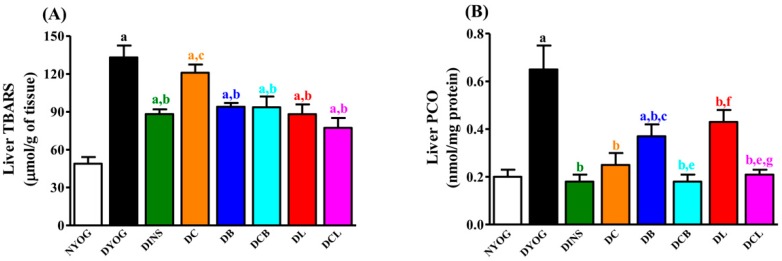
Hepatic levels of TBARS (**A**); and carbonyl protein groups (**B**) of STZ-diabetic rats after 50 days of treatment with yoghurt enriched with curcumin and carotenoids, individually or as mixtures. Values are expressed as means ± SEM, *n* = 10. Differences between groups were considered significant at *p* < 0.05 and were analyzed with one-way ANOVA followed by Student-Newman-Keuls test. a, different compared to NYOG; b, different compared to DYOG; c, different compared to DINS; d, different compared to DC; e, different compared to DB; f, different compared to DCB; g, different compared to DL (*p* < 0.05).

**Figure 4:**
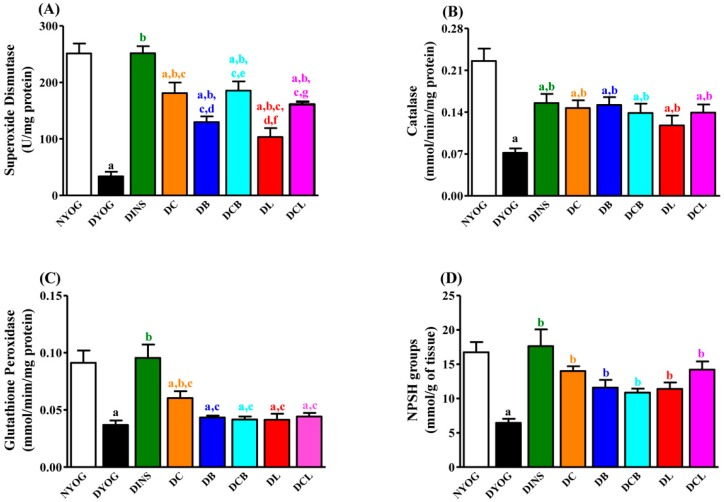
Hepatic activities of superoxide dismutase (SOD) (**A**); catalase (CAT) (**B**); glutathione peroxidase (GSH-Px) (**C**); and non-protein sulfhydryl groups (NPSH) levels (**D**) of STZ-diabetic rats after 50 days of treatment with yoghurt enriched with curcumin and carotenoids, individually or as mixtures. Values are expressed as means ± SEM, *n* = 10. Differences between groups were considered significant at *p* < 0.05 and were analyzed with one-way ANOVA followed by Student-Newman-Keuls test. a, different compared to NYOG; b, different compared to DYOG; c, different compared to DINS; d, different compared to DC; e, different compared to DB; f, different compared to DCB; g, different compared to DL (*p* < 0.05).

Oxidative stress was also very evident in the DYOG group, since biomarkers of lipid (thiobarbituric acid reactive substances, TBARS) and protein (PCO) oxidative damage were significantly increased in plasma (Figure 2B, TBARS) and liver (Figure 3, TBARS and PCO) compared to NYOG rats; ox-LDL levels were also increased in the plasma of DYOG rats (Figure 2C). In addition, significant losses in endogenous antioxidant defenses were observed in DYOG rats, since the activity of PON1 in plasma (Figure 2D) and the levels of hepatic antioxidant defenses (Figure 4) were decreased in DYOG rats compared to NYOG rats.

Finally, it should be pointed out that all of the metabolic disturbances observed in the STZ-diabetic rats were corrected with insulin treatment, demonstrating the responsiveness of this experimental model to pharmacological intervention. 

### 2.2. Body Weight, Adipose Tissue and Skeletal Muscle Weights and Physiological Parameters

Over 50 days, NYOG rats showed evident body weight gain when compared with the DYOG rats (Table 1); in addition, adipose tissue and skeletal muscle weights (Table 2) in the NYOG rats were higher than in DYOG, DC, DB, DCB, DL, and DCL rats. DYOG rats had the lowest terminal adipose tissue and skeletal muscle weights (Table 2), corroborating the minimal body weight gain (Table 1). Diabetic animals treated with insulin (DINS) had a body weight gain similar to that of the NYOG group (Table 1), and also similar terminal adipose tissue and skeletal muscle weights (Table 2).

After 50 days of treatment, diabetic rats treated with curcumin (DC) showed significant body weight gain when compared with DYOG rats (Table 1). Curcumin also promoted a significant increase in the weights of epididymal adipose tissue and *soleus* and EDL skeletal muscles (Table 2). In contrast, treatments of diabetic rats with bixin (DB) or lycopene (DL) did not alter body weight gain (Table 1) or change terminal adipose tissue and skeletal muscle weights compared with DYOG rats (Table 2).

After 50 days of treatment, co-administration of curcumin + bixin (DCB) or curcumin + lycopene (DCL) led to significant body weight gain, when compared with DYOG rats (Table 1), showing the benefit of these combined supplementations compared to carotenoids alone. In DCB and DCL rats, the weights of epididymal adipose tissues and *soleus* and EDL muscles (Table 2) were higher than in DYOG rats. It can be concluded that treatment with curcumin promotes an increase in body weight gain and leads to the preservation of adipose and muscle tissues, benefits that were maintained when curcumin was co-administered with bixin or lycopene.

After 50 days, the classical physiological symptoms of DM such as polyphagia, polydipsia, and polyuria were observed in DYOG rats, compared with day 0 and with NYOG rats (Table 1). As expected, treatment of diabetic rats with insulin (DINS group) prevented all these symptoms. Treatment with curcumin-enriched yoghurt diminished food and water intake, as well as the urinary volume of diabetic rats, although less efficiently when compared with insulin treatment (Table 1). Rats treated with bixin-enriched yoghurt (DB) or with lycopene-enriched yoghurt (DL) did not show changes in food and water intake or urinary volume compared with DYOG rats (Table 1).

Treatments with the mixtures curcumin + bixin (DCB) or curcumin + lycopene (DCL) were more effective compared with carotenoids alone; after 50 days of treatment, food and water intake and urinary volume were significantly decreased compared to DYOG rats (Table 1), thereby preserving the beneficial effects of curcumin on these altered physiological parameters.

### 2.3. Plasma Glucose Levels

DYOG rats showed a progressive increase in glycemia levels over the course of the study, evidence for the worsening of diabetes; in contrast, DINS rats had plasma glucose values near to normoglycemia (i.e. similar to NYOG group values), showing the effectiveness of insulin treatment in glucose correction and the responsiveness of this experimental model to antihyperglycemic intervention. Glycemia in NYOG rats remained within normal values until the end of experiment (Figure 1A).

Diabetic rats treated with curcumin-enriched yoghurt (DC) showed a decrease in fasting glycemia levels, reaching values similar those of DINS rats. Diabetic rats treated with yoghurt enriched with bixin (DB) or lycopene (DL) also had low glycemia levels in comparison with DYOG rats (Figure 1A). Although treatment with bixin reduced glycemia, the level of glycemia was still higher than in the NYOG, DINS, and DC groups. The glycemia levels of DB rats were also significantly higher after 40 days of treatment compared to day 0 (Figure 1A).

The treatments with curcumin + bixin (DCB) or curcumin + lycopene (DCL) were more effective in reducing glycemia than the carotenoid treatments alone; DCB and DCL rats had glycemia levels close to the value in DC rats (Figure 1A). Again, it can be concluded that co-administration of curcumin + lycopene or curcumin + bixin benefited glycemic control in diabetic rats better than the individual carotenoids, especially in the case of bixin.

### 2.4. Lipid Profile

The NYOG group had fasting levels of triacylglycerol (Figure 1B), total-cholesterol (Figure 2C), and HDL (Figure 2A) within the normal range. DYOG rats showed profound changes in lipid metabolism biomarkers. There was a progressive increase in triacylglycerol levels throughout the experimental period (Figure 1B), as well as elevated total-cholesterol levels (Figure 1C) and low HDL levels by the 50th day of the experiment (Figure 2A). Treatment with insulin (DINS) was effective in reducing the triacylglycerol and total-cholesterol levels (Figure 1B,C, respectively) to values similar those seen in NYOG rats. However, HDL levels were unchanged in diabetic rats treated with insulin, which remained low after 50 days of treatment (Figure 2A).

Low fasting triacylglycerol (Figure 1B) and total-cholesterol (Figure 1C) levels were observed in DC rats, from the 10th day of the treatment onward, with values being similar to those found in NYOG and DINS rats. However, the HDL levels were unchanged in diabetic rats treated with curcumin-enriched yoghurt (Figure 2A).

Treatment of diabetic rats with lycopene (DL) prevented the increase in triacylglycerol levels, which remained low from the 10th day of treatment onwards (Figure 1B). Treatment of diabetic rats with bixin (DB) caused a significant reduction in triacylglycerol levels but only after 40 days of treatment (Figure 1B). Total-cholesterol levels were significantly decreased in DB and DL groups, from the 10th day of treatment onwards (Figure 1C). Interestingly, HDL levels were increased in diabetic rats treated with lycopene (by 33%) or bixin (by 34%), compared to DYOG rats (Figure 2A).

Co-administration of curcumin and carotenoids maintained the beneficial effects of the individual treatments. In particular, two effects were of special significance: (i) the beneficial effects of curcumin on reducing triacylglycerol levels were maintained in the treatment with curcumin + bixin (Figure 1B); (ii) the mixtures of curcumin + lycopene or curcumin + bixin maintained the beneficial effects of the carotenoids in promoting an increase in the HDL levels in diabetic rats (Figure 2A).

### 2.5. Plasma Oxidative Stress Biomarkers 

After 50 days of diabetes, there was clear evidence for oxidative stress in DYOG rats; compared with NYOG rats, the plasma levels of TBARS (Figure 2B) and ox-LDL (Figure 2C) were increased, whereas the PON1 activity was decreased (Figure 2D). Treatment with insulin virtually prevented this oxidative stress.

Treatment with curcumin-enriched yoghurt decreased the levels of TBARS (43%) and ox-LDL (32%) in plasma of diabetic rats, compared with DYOG rats (Figure 2B,C, respectively); it also increased the activity of PON1 (32%, Figure 2D).

While bixin was unable to reduce the plasma levels of TBARS in diabetic rats, treatment with lycopene reduced plasma TBARS by 36%, compared to DYOG rats (Figure 2B). Plasma ox-LDL levels were also significantly decreased after treatment with bixin (38%) or lycopene (48%), compared with the DYOG group (Figure 2C). Treatment with bixin or lycopene also increased the activity of PON1 (29% and 30%, respectively, Figure 2D), compared with the DYOG group.

Treatment with the mixtures curcumin + bixin (DCB) or curcumin + lycopene (DCL) also provided benefit against lipoperoxidation, maintaining the best effects achieved by the treatments; the reduction in plasma TBARS in the DCB and DCL groups was similar to those observed in DC and DL rats (Figure 2B). With respect to ox-LDL levels, treatment with mixtures led to combined effects. The ox-LDL levels in diabetic rats from the DCB and DCL groups were further decreased in comparison to the decrease already observed in DC and DB rats (Figure 2C). The activity of PON1 was increased in DCB and DCL rats (38% and 36%, respectively, Figure 2D), in comparison with DYOG rats.

### 2.6. Liver Oxidative Stress Biomarkers

The onset of oxidative stress in diabetic rats was also evident at the level of the liver; the hepatic levels of TBARS (Figure 3A) and protein carbonyl groups (PCO) (Figure 3B) were clearly increased in the DYOG group, when compared with the NYOG group. Treatment with insulin was effective in preventing oxidative stress in the liver of diabetic rats.

Although the TBARS levels were not changed in the liver of DC rats (Figure 3A), treatment with curcumin-enriched yoghurt decreased hepatic PCO levels (61%), compared with DYOG rats (Figure 3B). Treatment of diabetic rats with individual carotenoids, bixin (DB) or lycopene (DL), promoted significant decreases in the hepatic levels of TBARS (29% and 34%, respectively, Figure 3A) and PCO (43% and 38%, respectively, Figure 3B) compared with DYOG rats. Liver TBARS in DCB and DCL rats were significantly decreased to levels similar those in the DB and DL groups (Figure 3A). The PCO levels were further decreased in rats from DCB and DCL groups, when compared with the DB and DL groups, respectively (Figure 3B).

### 2.7. Liver Antioxidant Activity

The activities of the antioxidant enzymes superoxide dismutase (SOD), catalase (CAT), and glutathione peroxidase (GSH-Px) (Figure 4A–C, respectively), as well as the non-protein sulfhydryl groups (NPSH) levels (Figure 4D) were decreased in the liver of DYOG rats compared with NYOG rats. Treatment with insulin increased the activities of these antioxidant enzymes as well as the NPSH levels in the liver of diabetic rats.

Treatments of diabetic rats with yoghurt enriched with the natural antioxidants, individually or as mixtures, showed benefits to the endogenous antioxidant systems. After 50 days, treatment with curcumin-enriched yoghurt increased the activities of SOD (approximately five-fold, Figure 4A), CAT (104%, Figure 4B), and GSH-Px (64%, Figure 4C) as well as the levels of NPSH (approximately two-fold, Figure 4D), when compared with DYOG rats. Compared with the DYOG group, treatment with bixin or lycopene also increased the activities of SOD (approximately three- and two-fold, respectively, Figure 4A) and CAT (111% and 66%, respectively, Figure 4B) and the NPSH levels (80% and 77%, respectively, Figure 4D), although these treatments showed no improvements in GSH-Px activity (Figure 4C).

Co-administration of curcumin and carotenoids produced combined effects on the increase in SOD activity: DCB and DCL rats had additional increases in the SOD activity compared with DB and DL rats, respectively (Figure 4A), maintaining the benefit provided by curcumin alone. CAT activity was increased in the DCB and DCL groups to a similar magnitude as all other treatments (Figure 4B); the activity of GSH-Px was not changed after treatment with the mixtures (Figure 4C). NPSH levels were increased in DCB and DCL groups to a similar magnitude as the DB and DC groups, respectively (Figure 4D).

Table 3 summarizes the beneficial effects of treatment with curcumin, bixin, and lycopene, individually or in mixtures, on the plasma and liver biomarkers of carbohydrate and lipid metabolism, oxidative damage, and antioxidant defenses, with an emphasis on the combined effects observed when the antioxidants are used as mixtures.

## 3. Discussion

This study provides evidence that in STZ-diabetic rats, treatment with yoghurt enriched with curcumin and carotenoids (lycopene or bixin) improved various biomarkers related to oxidative stress and cardiovascular risk. As far as we know, this study is the first to demonstrate two important findings about the in vivo benefits of a combination therapy based on these natural compounds for the management of DM. The ox-LDL levels of diabetic rats chronically treated with a combination of curcumin and lycopene or bixin decreased below the levels found in the individual treatments, reaching values similar those of non-diabetic animals. Co-administration of curcumin and carotenoids increased the HDL levels of diabetic rats, aggregating value to the beneficial effects of curcumin-enriched yoghurt (Table 3). Previous studies have shown that yoghurt enriched with curcumin alone improved various parameters in diabetic rats; however, no changes were observed in the HDL levels [19,20]. Based on this, it is suggested that a combination of curcumin and these carotenoids in yoghurt has great potential to protect diabetic individuals against long-term complications related to CVD.

The treatment with yoghurt alone did not cause any beneficial effect on the studied parameters in diabetic rats, which was an expected finding, since Gutierres et al. [19] previously observed that treatment of STZ-diabetic rats with yoghurt did not change the physiological and biochemical parameters, values remaining similar to those of diabetic rats receiving water (also via gavage). The beneficial effects of yoghurt alone against DM were not observed, probably due to the severity of the experimental model, and/or due to the low volume of yoghurt administered per day. However, fortuitously, the administration of curcumin and carotenoids using yoghurt solved the problem of the low solubility of these compounds in water; additionally, administration of these natural compounds in yoghurt did not impair their biological actions. Studies in collaboration are ongoing to investigate the effects of the yoghurt alone compared with yoghurt enriched with curcumin, lycopene, or bixin, in experimental animal models of obesity and insulin resistance (high-fat diet), which may be more suitable for the study of the possible beneficial roles of yoghurt alone. One of these possible yoghurt effects could be related to gut microbiota. Changes in the composition and diversity of the gut microbiota have been often observed in both obese mice and humans, mainly causing an increase in the *Firmicutes*:*Bacteroidetes* ratio [26]. It has been demonstrated that supplementation with probiotics beneficially alters the composition of gut microbiota, improving the interactions between gut microbes and host metabolism in obesity and other metabolic disorders [27,28]. Consequently, improvements in glucose and lipid metabolism and attenuation of the inflammatory and oxidative status have been observed in diabetic or obese individuals after yoghurt supplementation [29].

Studies showing the antidiabetic and antioxidant activities of individual treatment with lycopene or bixin have been reported. The beneficial effects of lycopene in DM have been consistently related to its antioxidant potential, attenuating endothelial dysfunction via reduction of both the oxidative stress in the aorta and in the levels of ox-LDL [22]. Recently, Ozmen et al. [23] observed that treatment of STZ-diabetic rats with lycopene reduced both vacuolization of the islets of Langerhans and the loss of insulin-secreting cells, leading to a fall in blood glucose levels compared with untreated diabetic rats; the authors attributed the effects of lycopene to its antioxidant activity. Corroborating these findings, the present study showed that treatment of diabetic rats with lycopene-enriched yoghurt reduced glycemia (Figure 1A), triacylglycerol levels (Figure 1B), and markers of oxidative damage (Figure 2 and Figure 3), as well as increased activities of PON1 (Figure 2D), SOD, and CAT (Figure 4A,B), as well as the levels of NPSH (Figure 4D). Cholesterol metabolism was also improved after lycopene supplementation; diabetic rats treated with lycopene-enriched yoghurt had low cholesterol plasma levels compared to untreated diabetic rats. HDL levels were also increased after lycopene treatment (Figure 2A). Evidence regarding the beneficial effects of lycopene on cholesterol metabolism have also been reported. According to the review of Palozza and collaborators [30], various mechanisms could explain the beneficial effects of lycopene on cholesterol metabolism: inhibition of the expression and activity of 3-hydroxy-3-methylglutaryl coenzyme A reductase (HMG-CoA reductase, a key enzyme in cholesterol synthesis); inhibition of the synthesis of the LDL receptor (thereby protecting cells from excessive cholesterol accumulation); inhibition of the activity of acyl-coenzyme A:cholesterol acyltransferase (ACAT, thereby preventing cholesterol ester accumulation in cells); increases in the expression of ATP-binding cassette ABC proteins (ABCA1, thereby increasing cholesterol efflux); increases in the circulating levels of the anti-atherogenic HDL. Taken together, the antioxidant capacity of lycopene and the beneficial effects on plasma cholesterol can be corroborated with the reduction observed in ox-LDL levels (Figure 2C), reiterating the potential role of lycopene in mitigating the CVD risk.

Few studies on the effects of bixin on DM symptoms have been performed; however, the findings are promising. Roehrs et al. [21] found that treatment of STZ-diabetic rats for 30 days with 10 or 100 mg/kg bixin caused a reduction in plasma levels of glucose, triacylglycerol, and cholesterol; treatment with bixin also reduced a marker of plasma protein oxidative damage and increased SOD activity, without changes in CAT and GSH-Px activities. In agreement with this, our data showed that STZ-diabetic rats treated with bixin-enriched yoghurt had low plasma levels of glucose (Figure 1A) and triacylglycerol (Figure 1B), although the lycopene effects on these biomarkers were more pronounced. The reduction in cholesterol promoted by bixin (Figure 1C) was similar to the lycopene effect. Bixin-enriched yoghurt also decreased the hepatic levels of thiobarbituric acid reactive substances (TBARS) and protein carbonyl groups (PCO) (Figure 3) and increased the activities of PON1 (Figure 2D), SOD, and CAT (Figure 4A,B) as well as NPSH levels (Figure 4D). The reduction in ox-LDL levels (Figure 2C) and the increase in HDL levels (Figure 2A) after the treatment of diabetic rats with yoghurt-enriched bixin were similar to the lycopene effects. Although the individual treatments with lycopene and bixin promoted similar responses in most of the parameters, the differences in the magnitude of the responses in glycemia and triacylglycerol between these carotenoids (for example, lycopene was better than bixin) can be explained by the fact that polar carotenoids (such as bixin) have a rapid clearance rate from the plasma [31]; hence, if bixin also has a short half-life, this could possibly be the reason why the TBARS levels were not decreased in the plasma of diabetic rats treated with bixin (Figure 2B).

The antidiabetic and antioxidant activities of curcumin have been well documented [19,20], as well as potential mechanisms explaining its antidiabetic activity. Although the stimulation of insulin release from pancreatic beta bells has been cited as one mechanism by which curcumin exerts its antihyperglycemic effect [32], it is unlikely that the treatment with curcumin-enriched yoghurt is able to prevent the loss of pancreatic function in STZ-diabetic rats. Junod et al. [33] found that, 24 hours after the intravenous administration of STZ in doses up to 40 mg/kg, Wistar rats develop symptoms of type 1 DM, such as hyperglycemia, glycosuria, and significant decreases in pancreatic and serum insulin; these metabolic derangements remained unchanged for long periods after STZ administration. According to Gutierres et al. [34], the anti-hyperglycemic effect of curcumin-enriched yoghurt may be related to its ability to increase both peripheral insulin sensitivity and glucose tolerance in STZ-diabetic rats; in *gastrocnemius* skeletal muscles, this seems to be associated with an increase in AKT phosphorylation and GLUT4 translocation. These mechanisms may explain, at least in part, the reduction in glycemia (Figure 1A) in diabetic rats treated with curcumin, thereby improving physiological parameters, such as body and tissue weight gain, food and water intake, and urinary volume (Table 1 and Table 2).

Chronic hyperglycemia in DM accounts for the establishment of oxidative stress in many tissues (liver, kidney, retina, and peripheral nerves) due to an overproduction of ROS; so the anti-hyperglycemic effect of curcumin-enriched yoghurt explains, at least in part, the reduction in the levels of the oxidative stress biomarkers (ox-LDL, Figure 2C; TBARS and PCO, Figure 3A,B) and the increase in PON1 (Figure 2D), SOD, CAT, and GSH-Px antioxidant enzymes (Figure 4A–C) and NPSH levels (Figure 4D) in STZ-diabetic rats. However, curcumin also possesses the ability to scavenge ROS [35,36] and to inhibit LPO [37], also corroborating the in vivo antioxidant activity. However, although curcumin improved various parameters in diabetic rats, no improvement was observed in HDL levels (Figure 2A); in addition, the beneficial effects of curcumin against diabetic disturbances did not reach the magnitude of response obtained with insulin treatment. Together, these facts have prompted us to study the combination of curcumin with other natural compounds that could contribute additional benefits in the treatment of DM.

The findings of the present study showed that the treatment with mixtures of curcumin + lycopene or curcumin + bixin caused a combination effect on the reductions in glycemia (Figure 1A), triacylglycerol (Figure 1B), PCO levels (Figure 3B), and in the increase of SOD activity (Figure 4A), maintaining the beneficial effect of the individual treatment (i.e., curcumin). The combined effects of the mixtures were also observed in the reduction of ox-LDL levels (Figure 2C), a central risk factor for CVD. Furthermore, mixtures were able to increase HDL levels (Figure 2A), a beneficial effect attributed to lycopene and bixin alone and which was maintained after combining either of them with curcumin.

Treatments with the individual carotenoids, or in mixtures with curcumin, were not able to increase the activity of GSH-Px (Figure 4C). It was a question if the combination between the increases in the activities of SOD and CAT (Figure 4A,B) and the intrinsic antioxidant potential of the carotenoids would be sufficient to reduce oxidative stress in the liver of diabetic rats. This question was answered in the positive by the observation of decreased levels of TBARS and PCO in the liver (Figure 3A,B) and by the restoration of NPSH levels (Figure 4D).

Carotenoids are transported into the circulation mostly in association with lipoproteins. It has been demonstrated that lycopene and bixin are transported predominantly in HDL and LDL [38,39]. Apart from the lipoprotein fraction carrying the carotenoids, both lycopene and bixin were able to protect LDL against oxidation to a magnitude comparable to that seen with curcumin (Figure 2C). It is possible that LDL is protected directly against oxidative damage, at least in part, by the antioxidant capacity of the carotenoids transported in the lipoprotein. In addition, the anti-hyperglycemic effect of carotenoids (Figure 1A) cannot be dismissed as a protective mechanism against LDL oxidation. Curcumin seems to prevent LDL oxidation by a mechanism independent of transport by lipoproteins, since it is carried by albumin in the circulation [40]. Furthermore, the curcumin antioxidant capacity is also preserved when it is coupled to albumin [41]. 

The combination effect of lycopene with other natural antioxidants resulting in protection of LDL from oxidation has been previously observed in an elegant in vitro study by Fuhrman et al. [15]. Using lipoprotein oxidation stimulated by incubation in the presence of AAPH or copper ions, it was postulated by the authors that lycopene acts synergistically with natural antioxidants (vitamin E, glabridin, rosmarinic acid, carnosic acid) as an effective antioxidant against LDL oxidation. However, curcumin and carotenoids have distinct antioxidant mechanisms; while curcumin is able to donate a H-atom from the phenolic group as well as from the central methylenic bridge in the hepta-dienone moiety and has a marked capacity for iron binding [35,42], lycopene (and possibly bixin) prevents lipid peroxidation via singlet oxygen quenching [43] or scavenging of peroxyl radicals [44]. The combined effects of curcumin and carotenoids in preventing LDL oxidation was so effective that the ox-LDL levels of DCB and DCL rats were similar to those levels found in NYOG and DINS rats (Figure 2C). 

In addition to the prevention of LDL oxidation in diabetic rats, the combination of curcumin with carotenoids also showed benefits against cardiovascular complications via an increase in HDL levels (Figure 2A). The increase in circulating HDL and/or the improvement in its antioxidant functionality have been pointed to as a central strategy associated with effective therapeutic interventions having antiatherogenic properties; the antioxidant potential of HDL has been related to PON1 [45].

PON1 mostly circulates in association with HDL; its antioxidant activity is associated with its ability to hydrolyze lipid peroxides [46]. In addition to protecting HDL against oxidative damage [47], it has been consistently shown that PON1 dampens LDL oxidation [48]. The present study showed that STZ-diabetic rats had diminished PON1 activity (Figure 2D), as has previously been observed in other studies, in both humans [49] and rodents [50] with diabetes. Treatment with insulin virtually prevented the decrease in PON1 activity in diabetic rats, without improving HDL levels. Modification of PON1 by glycation inhibits its activity towards paraoxon [51,52]; therefore, the antihyperglycemic effect of insulin may explain its ability to avoid the fall in the PON1 activity due to DM. Treatment of diabetic rats with curcumin and carotenoids, individually or as mixtures, increased the activity of PON1 (Figure 2D). It has been consistently demonstrated that various natural antioxidants, including curcumin [53], can increase the activity of PON1 via upregulation of its expression in liver [54]. It has been also shown that lycopene supplementation increases the PON1 activity in association with an increase in HDL levels [55]. Furthermore, considering the ability of PON1 to protect LDL against oxidation, the decrease in the ox-LDL levels of diabetic rats treated with curcumin + lycopene or curcumin + bixin could be a consequence of the increase in both HDL levels and PON1 activity. Taken together, the bulk of improvements observed in various biomarkers related with cardiovascular events support the antiatherogenic potential of mixtures of curcumin and carotenoids.

## 4. Material and Methods

### 4.1. Animals

Male Wistar rats (*Rattus norvegicus*) weighing 150 ± 10 g (6 weeks) were housed in individual metabolic cages, under controlled conditions of temperature (23 °C ± 1 °C) and humidity (55% ± 5%) with a 12 h light/dark cycle. Rats received water and lab chow diet (Presence, Paulínia, São Paulo, Brazil) *ad libitum* throughout the 50 days of experiment. The experimental procedures were approved by the Committee for Ethics in Animal Experimentation from the School of Pharmaceutical Sciences, UNESP, Araraquara, SP (CEUA/FCF/CAr resolution number 44/2013, 15 August 2013).

### 4.2. Induction of Experimental Diabetes Mellitus

After an acclimation period, experimental type 1 diabetes mellitus was induced by a single intravenous injection of 40 mg/kg streptozotocin (STZ, Sigma Aldrich, St. Louis, MO, USA) dissolved in 0.01 M citrate buffer (pH 4.5), in previously 12 h fasted rats. Normal rats received only citrate buffer. For this procedure, all animals were anesthetized with isoflurane. Three days after STZ administration, rats with post-prandial glycemia values of approximately 450 mg/dL were used as the diabetic groups [20,56]. Glycemia levels were determined using the glucose oxidase method [57] using a commercial kit (Labtest Diagnostica SA, Lagoa Santa, Minas Gerais, Brazil). Diabetic animals were sorted into the different experimental groups using matched values of hyperglycemia and body weight.

### 4.3. Experimental Design and Treatment 

Extracts of *Curcuma longa* rhizomes (65% curcumin, Sigma Aldrich, St. Louis, MO, USA), *Bixa orellana* seeds (60% bixin, Lychnoflora, Ribeirão Preto, São Paulo, Brazil) or tomato (10.13% lycopene, PHD Com. Imp. Exp. LTDA, São Paulo, São Paulo, Brazil) were mixed with commercial plain yoghurt (170 g contained 9.1 g carbohydrates, 6.8 g protein, 7.0 g total fat, 126 kcal, Nestlé, Brazil) using a homogenizer (27,000 rpm) for 90 seconds at a controlled ambient temperature (25 °C).

Diabetic rats were sorted into seven groups (10 rats/group) as follows: diabetic rats treated with yoghurt (DYOG); 90 mg/kg curcumin in yoghurt (DC); 5.5 mg/kg bixin in yoghurt (DB); 90 mg/kg curcumin + 5.5 mg/kg bixin (DCB) in yoghurt; 45 mg/kg lycopene in yoghurt (DL); 90 mg/kg curcumin + 45 mg/kg lycopene (DCL) in yoghurt; 4U insulin (DINS). A group of normal rats was also treated with yoghurt (NYOG). The curcumin dose was chosen based on previous studies from our laboratory [19,20]. Previous pilot experiments allowed us to choose the minimally effective dose of each of the carotenoids in promoting beneficial effects against the metabolic disturbances in the STZ-diabetic rats. Considering the purity of each extract and the established daily doses, each treated group received the following quantity of each natural antioxidant: DC, DCB, and DCL rats received 58.5 mg/kg/day curcumin; DB and DCB rats received 3.3 mg/kg/day bixin; DL and DCL rats received 4.5 mg/kg/day lycopene. In addition, it must be emphasized that animals treated with extracts containing bixin or lycopene received the same daily quantity of carotenoid (8.4 μmol). The treatments, except for DINS, were performed by gavage, twice a day with a half-dose, at 08:00 h and 17:00 h, for a total of 50 days. The half-doses of the natural compounds were administered in 0.5 mL of yoghurt, giving a total dose of yoghurt of 1.0 mL/rat/day. DINS rats received two subcutaneous injections of insulin, 2U/rat for each injection, at 08:00 h and 17:00 h, over the 50-day course of the experiment. Fifty days of treatment has previously been reported to be the minimal treatment time required to observe the beneficial effects of these natural antioxidants on reducing or preventing the metabolic disturbances caused by experimentally induced diabetes mellitus, especially those related to oxidative stress [20].

Every 10 days, prior to blood collection to measure plasma parameters, animals were fasted for 12 h in order to minimize the interference of food intake in the results of lipid profile. Blood was collected from the tail-tip of animals into heparinized micro-tubes (Hemofol, Itapira, São Paulo, Brazil; 5000 UI/mL), after a peripheral vasodilatation. The blood was centrifuged for 10 min at 2500 rpm and the plasma obtained was collected and used for biochemical measurements.

Every 10 days, body weight, food intake (12 h), water intake, and urinary volume (24 h) were assessed along with measurements of the plasma levels of glucose, triacylglycerol, and total-cholesterol, using commercial kits from Labtest Diagnostica SA (Lagoa Santa, Minas Gerais, Brazil). After 50 days of treatment, the rats were euthanized by decapitation, and blood samples were used for the determination of the aforementioned plasma biochemical parameters. In addition, plasma samples were used for the measurement of HDL-cholesterol (HDL) (kits from Labtest Diagnostica SA, Lagoa Santa, Minas Gerais, Brazil), thiobarbituric acid reactive substances (TBARS), and oxidized LDL (ox-LDL). The epididymal and retroperitoneal white adipose tissues and the *soleus* and *extensor digitorum longus* (EDL) skeletal muscles were removed and weighed. A piece of the liver was also removed and frozen (−80 °C) for the subsequent analysis of biomarkers of oxidative damage, activities of antioxidant enzymes, and non-protein sulfhydryl group (NPSH) levels.

### 4.4. Oxidative Stress Biomarkers

#### 4.4.1. Lipid Peroxidation (LPO)

Liver samples (0.25 g) were homogenized in 1 mL of 1.15% potassium chloride at 4 °C. The homogenates were centrifuged at 10,000× *g* for 10 min at 4 °C and the supernatants were used for analysis. Plasma and liver supernatants were previously deproteinized according to Pilz et al. [58]. LPO diene end-products, including malondialdehyde (MDA), were measured using a thiobarbituric acid (TBA) reaction [59]. Thiobarbituric acid reactive substances levels were measured spectrophotometrically at 535 nm (liver) or fluorometrically with excitation and emission wavelengths of 510 and 553 nm, respectively (plasma); 1,1,3,3-tetramethoxypropane (Sigma Aldrich, St. Louis, MO, USA) was used as standard. The results were expressed as µmol/L/g tissue (liver) or µmol/L of the plasma thiobarbituric acid reactive substances.

#### 4.4.2. Protein Carbonyl Groups (PCO)

The PCO levels in liver were determined according to Levine et al. [60]. Carbonyl groups in proteins react with 2,4-dinitrophenylhydrazine to form 2,4 dinitrophenylhydrazone, which was monitored spectrophotometrically at 370 nm. The concentration of PCO was obtained using the molar extinction coefficient of the hydrazone (22,000 M^−1^·cm^−1^). Results were expressed as µmol/mg protein.

#### 4.4.3. Oxidized LDL (ox-LDL)

Plasma levels of oxidized LDL were measured by enzyme immunoassay (ELISA) (Uscn Life Science Inc., Wuhan, China). The results were expressed as ng/mL.

#### 4.4.4. Non-Protein Sulfhydryl (NPSH) Groups

Non-protein sulfhydryl groups (NPSH) represent an indirect measurement of GSH and were determined according to Sedlak and Lindsay [61], by measuring the reduction of 5,5-dithiobis-(2-nitrobenzoic acid) at 412 nm. Results were expressed as mmol/L/g tissue (liver).

#### 4.4.5. Paraoxonase 1 (PON1) Activity

PON1 activity was determined according to Costa et al. [62], with modifications. Plasma PON1 activity was measured by the hydrolysis of paraoxon and release of *p*-nitrophenol. 

The assay mixture contained 10 µL of plasma in 15 mM Tris/HCl buffer (pH 8.5) containing 0.15 mM CaCl_2_, 0.3 M NaCl, and 1.2 mM of freshly prepared paraoxon. The stock solution of 120 mM paraoxon was prepared in acetone and kept on ice; before use, this stock solution was diluted 1:40 in water and its concentration was monitored at 274 nm, using the molar extinction coefficient of paraoxon in water (8900 M^−1^·cm^−1^) [63].

The assay was performed at 37 °C and initiated by the addition of paraoxon; the activity was monitored by measuring absorbance at 405 nm over a period of 5 min. The results were calculated assuming the molar extinction coefficient of *p*-nitrophenol (18,000 M^−1^·cm^−1^, [64]. The PON1 activity was expressed in units/liter (unit = µmoL paraoxon hydrolyzed/min).

### 4.5. Activities of the Antioxidant Enzymes in Liver

#### 4.5.1. Sample preparation

Liver samples (0.1 g) were homogenized in 1 mL of sodium phosphate buffer (10 mmol/L, pH 7.4) at 4 °C. The homogenates were centrifuged at 10,000× *g* for 10 min at 4 °C and the supernatants were used for the analysis of the activities of SOD, CAT, and GSH-Px. Protein levels in the supernatants were determined according to Lowry et al. [65], using bovine serum albumin as standard.

#### 4.5.2. Superoxide Dismutase (SOD) Activity

SOD activity was evaluated according to Beauchamp and Fridovich [66]; the oxidation of xanthine generates superoxide anion (O_2_^•−^) by the catalytic action of xanthine oxidase, which reduces nitroblue tetrazolium chloride (NBT) to a formazan product. In the presence of SOD, inhibition of NBT reduction occurs, which is monitored at 550 nm. The results were expressed as U/mg protein. One unit of SOD is defined as the enzyme amount required to inhibit the rate of NBT reduction by 50%.

#### 4.5.3. Catalase (CAT) Activity

CAT activity was measured by monitoring the disappearance of hydrogen peroxide (H_2_O_2_) at 230 nm [67]. The results were expressed as mmol of H_2_O_2_ consumed/min/mg protein.

#### 4.5.4. Glutathione Peroxidase (GSH-Px) Activity

GSH-Px activity was evaluated according to Rush and Sandiford [68]. GSH-Px catalyzes the oxidation of GSH in the presence of H_2_O_2_. In the presence of gluthatione reductase, the oxidized gluthatione is reduced to GSH with concomitant oxidation of NADPH to NADP^+^. NADPH disappearance was monitored at 340 nm. The results were expressed as mmol of NADPH consumed/min/mg protein.

### 4.6. Statistical Analysis

Data are expressed as mean ± standard error of mean (SEM). Statistical analysis was performed using one-way analysis of variance (ANOVA) followed by Student-Newman-Keuls test to compare the temporal inter-group differences in body weight and in biochemical and oxidative stress biomarkers. A paired Student’s *t*-test was used to compare the intra-group changes in the parameters relative to day 0. Data were considered statistically different at *p* < 0.05. Statistical analyses were performed using the program Graphpad Instat (GraphPad Software, 3.05 version, La Jolla, CA, USA).

## 5. Conclusions

The findings of the present study provided evidences for the combined effects of yoghurt enriched with curcumin in mixtures with lycopene or bixin, bringing various benefits for the treatment of the wide range of diabetic disturbances. In summary, while curcumin contributes to the beneficial effects on both carbohydrate and lipid metabolism and oxidative stress biomarkers, the carotenoids increased high-density lipoprotein (HDL) levels and also diminished biomarkers of oxidative damage. When administered as mixtures, the combined effects of curcumin and carotenoids aggregated benefits toward the treatment of various disturbances in streptozotocin (STZ)-diabetic rats. The combined effects led to prevention of low-density lipoprotein (LDL) oxidation, which can be associated with an increase in HDL levels and paraoxonase 1 (PON1) activity, thereby lowering these cardiovascular risk factors in diabetes.

Oxidative stress is one mechanism that participates in the establishment of microvascular [69] and macrovascular [70] complications even after maintenance of normoglycemia under intensive antihyperglycemic treatment, a phenomenon often referred to as “metabolic memory.” In front of this, complementary therapies targeting not only the hyperglycemia but also other metabolic disturbances in diabetes mellitus (DM), such as oxidative stress, are appearing as promising strategies in combating diabetic complications [71].

Taken together, the findings of this study support the need for ongoing investigations into the use of curcumin mixed with carotenoids into yoghurt as a food supplementation with anti-atherogenic potential.

## Figures and Tables

**Table 1:** Body weight and food intake of streptozotocin (STZ)-diabetic rats before (day 0) and after (day 50) treatment with yoghurt enriched with curcumin and carotenoids individually or in mixtures.

Physiological Parameters	Groups															
	NYOG		DYOG		DINS		DC		DB		DCB		DL		DCL	
Parameters	Day 0	Day 50	Day 0	Day 50	Day 0	Day 50	Day 0	Day 50	Day 0	Day 50	Day 0	Day 50	Day 0	Day 50	Day 0	Day 50
**Body weight (g)**	134.07 ± 8.03	319.32 ± 20.28 (#)	131.56 ± 2.11	204.10 ± 10.86 (a,#)	126.90 ± 2.88	308.00 ± 10.66 (b,#)	130.43 ± 1.72	281.90 ± 6.49 (a,b,#)	130.14 ± 3.83	234.83 ± 17.45 (a,c,#)	132.13 ± 3.79	277.50 ± 11.12 (a,b,#)	131.00 ± 3.44	246.10 ± 18.56 (a,c,#)	133.00 ± 2.10	279.70 ± 10.17 (a,b,#)
**Food intake (g/12 h)**	5.75 ± 1.40	9.50 ± 1.59	7.69 ± 0.84	22.00 ± 1.74 (a,#)	7.33 ± 0.85	8.00 ± 0.68 (b)	7.11 ± 0.48	12.83 ± 2.12 (b,#)	7.60 ± 1.89	17.25 ± 1.29 (a,c,#)	7.33 ± 0.88	13.67 ± 0.44 (b,#)	7.58 ± 1.29	15.44 ± 2.18 (a,c,#)	7.92 ± 0.37	12.70 ± 1.90 (b,#)
**Water intake (mL/24 h)**	5.14 ± 1.75	17.00 ± 2.60 (#)	39.11 ± 5.30	91.47 ± 9.68 (a,#)	40.40 ± 8.42	18.83 ± 2.93 (b,#)	42.20 ± 4.62	52.20 ± 9.20 (b)	39.00 ± 7.03	60.29 ± 9.80 (a,c)	33.11 ± 7.56	53.20 ± 4.31 (b,#)	42.00 ± 5.02	62.57 ± 9.30 (a,c)	45.40 ± 3.86	56.43 ± 7.96 (b)
**Urinary volume (mL/24 h)**	3.58 ± 2.18	16.17 ± 3.34 (#)	28.33 ± 3.12	108.33 ± 10.76 (a,#)	25.80 ± 6.98	28.00 ± 4.21 (b)	29.13 ± 2.16	54.80 ± 9.65 (a,b,#)	29.86 ± 4.90	67.11 ± 10.40 (a,c,#)	25.17 ± 4.74	57.17 ± 9.23 (a,b,#)	28.00 ± 3.47	70.00 ± 7.33 (a,c,#)	32.40 ± 1.66	56.33 ± 5.06 (a,b,#)

Values are expressed as means ± SEM, *n* = 10. Differences between groups were considered significant at *p* < 0.05 and were analyzed with one-way ANOVA followed by Student-Newman-Keuls test. a, different compared to NYOG; b, different compared to DYOG; c, different compared to DINS (*p* < 0.05). Different compared to the same group relative to day 0 were analyzed with a paired Student’s t test. #. different compared to day 0 (*p* < 0.05). Normal (N) and diabetic (D) rats treated with yoghurt (NYOG and DYOG, respectively) or yoghurt enriched with curcumin (DC), bixin (DB), lycopene (DL), curcumin + bixin (DCB) or curcumin + lycopene (DCL).

**Table 2:** Weights of white adipose tissues and skeletal muscles of STZ-diabetic rats after 50 days of treatment with yoghurt enriched with curcumin and carotenoids individually or in mixtures.

Tissues Weights	Groups							
	NYOG	DYOG	DINS	DC	DB	DCB	DL	DCL
**Epididymal adipose tissue (g)**	4.753 ± 0.478	1.226 ± 0.112 (a)	4.348 ± 0.2588 (b)	1.873 ± 0.119 (a,b,c)	1.603 ± 0.262 (a,c)	2.160 ± 0.140 (a,b,c)	1.522 ± 0.229 (a,c)	1.661 ± 0.033 (a,b,c)
**Retroperitoneal adipose tissue (g)**	4.517 ± 0.304	0.611 ± 0.170 (a)	3.949 ± 0.449 (b)	1.242 ± 0.180 (a,c)	0.768 ± 0.276 (a,c)	1.190 ± 0.136 (a,c)	0.808 ± 0.248 (a,c)	1.214 ± 0.186 (a,c)
***Soleus* muscle (g)**	0.155 ± 0.003	0.105 ± 0.007 (a)	0.163 ± 0.004 (b)	0.137 ± 0.004 (a,b)	0.109 ± 0.010 (a,c)	0.134 ± 0.007 (a,b)	0.107 ± 0.010 (a,c)	0.120 ± 0.005 (a,b)
**EDL muscle (g)**	0.151 ± 0.004	0.096 ± 0.006 (a)	0.153 ± 0.005 (b)	0.121 ± 0.004 (a,b)	0.095 ± 0.008 (a,c)	0.118 ± 0.004 (a,b)	0.093 ± 0.008 (a,c)	0.115 ± 0.008 (a,b)

Values are expressed as means ± SEM, *n* = 10. Differences between groups were considered significant at *p* < 0.05 and were analyzed with one-way ANOVA followed by Student-Newman-Keuls test. a, different compared to NYOG; b, different compared to DYOG; c, different compared to DINS (*p* < 0.05).

**Table 3:** Relative changes (% of DYOG) in plasma and liver biomarkers of carbohydrate and lipid metabolism, oxidative damage, and antioxidant defenses in STZ-diabetic rats after 50 days of treatment with yoghurt enriched with curcumin and carotenoids individually or in mixtures, with an emphasis on the effects when the antioxidants are in mixtures.

Biochemical Parameters	DC	DB	DL	DCB	DCL
**Plasma**					
Glycemia	↓ **63%**	↓ 32%	↓ 59%	↓ **67%**	↓ **66%**
Triacylglycerol	↓ **61%**	↓ 31%	↓ **61%**	↓ **60%**	↓ **62%**
Total-Cholesterol	↓ 21%	↓ 21%	↓ 22%	↓ 22%	↓ 23%
HDL-Cholesterol	no effect	↑ **34%**	↑ **33%**	↑ **32%**	↑ **33%**
TBARS	↓ **43%**	no effect	↓ 36%	↓ **39%**	↓ **44%**
PON1	↑ **32%**	↑ 29%	↑ 30%	↑ **38%**	↑ **36%**
ox-LDL	↓ **32%**	↓ 38%	↓ **48%**	↓ 55% *	↓ **58%**
**Liver**					
TBARS	no effect	↓ **29%**	↓ **34%**	↓ **30%**	↓ **42%**
PCO	↓ **61%**	↓ 43%	↓ 38%	↓ **72%**	↓ **68%**
SOD	↑ **434%**	↑ 282%	↑ 204%	↑ **447%**	↑ **375%**
CAT	↑ **104%**	↑ **111%**	↑ 66%	↑ **92%**	↑ **93%**
GSH-Px	↑ 64%	no effect	no effect	no effect	no effect

Values in “**Bold**” represent maintenance of the beneficial effect when the antioxidant was administered as mixtures. *ox-LDL levels were further decreased in DCB rats compared with the decrease in DC and DB rats. Normal (N) and diabetic (D) rats treated with yoghurt (NYOG and DYOG, respectively) or yoghurt enriched with curcumin (DC), bixin (DB), lycopene (DL), curcumin + bixin (DCB) or curcumin + lycopene (DCL).

## Abbreviations

AAPH	2′,2′-azobis(2-amidinopropane) hydrochloride
CAT	catalase
CVD	cardiovascular disease
DM	diabetes mellitus
EDL	*extensor digitorum longus*
GSH-Px	glutathione peroxidase
H_2_O_2_	hydrogen peroxide
HDL	high-density lipoprotein
LDL	low-density lipoprotein
LPO	lipid peroxidation
MDA	malondialdehyde
NADPH	nicotinamide adenine dinucleotide phosphate, reduced form
NBT	nitroblue tetrazolium chloride
NPSH	non-protein sulfhydryl groups
O_2_^•−^	superoxide anion radical
ox-LDL	oxidized LDL
PCO	protein carbonyl groups
PON1	paraoxonase 1
ROS	reactive oxygen species
SEM	standard error of mean
SOD	superoxide dismutase
STZ	streptozotocin
TBA	thiobarbituric acid
TBARS	thiobarbituric acid reactive substances
